# Abnormal Bone Metabolism May Be a Primary Causative Factor of Keel Bone Fractures in Laying Hens

**DOI:** 10.3390/ani11113133

**Published:** 2021-11-02

**Authors:** Haidong Wei, Yanqing Chen, Haoyang Nian, Jing Wang, Yilin Liu, Jianxing Wang, Kaiqi Yang, Qian Zhao, Runxiang Zhang, Jun Bao

**Affiliations:** 1 College of Animal Science and Technology, Northeast Agricultural University, Harbin 150030, China; weihaidongneau@163.com (H.W.); chenyanqing98@163.com (Y.C.); 18846076532@163.com (H.N.); liuyilin5693132@163.com (Y.L.); Katherine619kele@163.com (K.Y.); zhaoqian@neau.edu.cn (Q.Z.); 2 College of Life Science, Northeast Agricultural University, Harbin 150030, China; xiaojing050416@163.com (J.W.); wjx13473060303@163.com (J.W.); 3 Key Laboratory of Chicken Genetics and Breeding, Ministry of Agriculture and Rural Affairs, Harbin 150030, China

**Keywords:** laying hens, keel bone damage, bone metabolism, bone health, furnished cage

## Abstract

**Simple Summary:**

Keel is an essential structural bone, providing anchorage for the attachment of large breast muscles in birds, allowing them to flap wings and provide proper ventilation for their lungs during flight. Previous studies reported that keel bone damage (especially fractures) negatively affects the welfare, health, production performance, eggshell quality, and mobility of laying hens contained in different housing systems. Furthermore, various factors affect keel bone damage, including nutrition, age, housing systems, and strains of laying hens. However, studies on the effects of abnormal bone metabolism and development on keel bone damage in laying hens are limited. Therefore, this study aimed to investigate the impacts of bone metabolism and development status on keel bone damage by determining the levels of serum bone turnover markers in laying hens. The results showed that laying hens with impaired keel bone had significantly altered levels of serum Ca and P metabolism-related and osteoblast and osteoclast activity-related markers compared to those in laying hens with normal keel bone. Thus, these results indicated that abnormal bone metabolism before keel bone damage reflected by varying levels of serum bone turnover markers might be a pivotal factor causing keel bone damage in laying hens. Our results also provide new insights into the occurrence of keel bone damage in laying hens.

**Abstract:**

Keel bone damage negatively affects the welfare, production performance, egg quality, and mobility of laying hens. This study aimed to investigate whether abnormal bone metabolism causes keel bone damage in laying hens. Eighty Hy-line Brown laying hens were housed in eight furnished cages with 10 birds per cage and studied from 18 to 29 weeks of age (WOA). Accordingly, keel bone status was assessed at 18, 22, 25, and 29 WOA using the X-ray method, and the serum samples of laying hens with normal keel (NK), deviated keel (DK), and fractured keel (FK) that occurred at 29 WOA were collected across all the time-points. Subsequently, the serum samples were used to measure markers related to the metabolism of Ca and P and activities of osteoblast and osteoclast. The results showed that FK laying hens had lighter bodyweight than NK and DK birds throughout the trial (*p* < 0.05), while the keel bone length and weight were not different in NK, DK, and FK hens at 29 WOA (*p* > 0.05). Moreover, bone hematoxylin and eosin (H&E) staining and tartrate-resistant acid phosphatase (TRAP) staining indicated that damaged keel bone had evident pathological changes. In the FK hens, serum P level was reduced but serum 1,25-dihydroxy-vitamin D_3_ (1,25-(OH)_2_D_3_) and 25-hydroxyvitamin D_3_ (25-OHD_3_) levels were elevated compared to NK hens (*p* < 0.05). Additionally, DK hens had higher levels of serum 1,25-(OH)_2_D_3_, parathyroid hormone (PTH) and calcitonin (CT), and lower level of serum 25-OHD_3_ than the NK birds (*p* < 0.05). Furthermore, serum alkaline phosphatase (ALP), osteocalcin (OC), osteoprotegerin (OPG), TRAP, and corticosterone (CORT) levels were elevated in DK and FK hens compared to NK hens (*p* < 0.05). The levels of serum Ca, P, PTH, ALP, TRAP, OPG, OC, and CORT in laying hens fluctuated with the age of the birds. Generally, the results of this study indicate that keel bone damage, especially fractures, could be associated with abnormal bone metabolism in laying hens.

## 1. Introduction

Commercial laying hens are increasingly being housed in alternative housing systems, including furnished cages, aviaries, free-range, and free-run floor housing systems due to growing interest in animal welfare and the ban of conventional battery cages [1]. Keel bone damage (including fractures and deviations), however, is more prevalent and severe in all alternative laying hen production systems relative to conventional cages. Keel bone damage (especially fractures) does not only affect the emotional and physiological status and alters behavior, but also reduces the welfare, egg production, and egg quality in laying hens, and it has therefore become an important welfare and health issue for the poultry industry [2].

On the one hand, housing systems, breeds, age, and the nutrition of laying hens influence the incidence of keel bone fractures (KBFs) [3,4]. Previous studies on laying hen housing systems reported that KBF prevalence is higher in a single-tier floor housing system than a cage housing system [5], and that it is elevated in aviary and free-range systems compared to furnished cages [6]. Studies on strains found that commercial laying hens have higher susceptibility to KBFs than non-commercial birds [7], and brown laying hens have more KBFs prevalence in comparison to white strains [8]. A recent literature review by Toscano et al. [9] stated that the incidence of KBFs is increased with age, with a maximum during the peak period of laying, gradually reducing after 49 weeks of age (WOA) in laying hens. Additionally, other studies found that feeding laying hens with omega-3 fatty acids-enriched diets can decrease the incidence of KBFs [10,11]. Overall, these findings indicate that KBFs are multifactorial.

In addition, Casey-Trott et al. [12,13] reported that providing exercise opportunities during pullet rearing could improve overall bone composition and strength at 16 WOA, and reduce the prevalence of keel bone damage in Lohmann Selected Leghorn Lite laying hens during the entire laying period. Our recent study on Lohmann White laying hens at 42 WOA found that KBFs are associated with the differences in bone metabolism and remodeling-related markers, including the activities of alkaline phosphatase (ALP) and tartrate-resistant acid phosphatase (TRAP), and the contents of calcium (Ca) and phosphorus (P) in serum and bones [14]. These results suggested that keel bone damage might be related to bone metabolism. Serum and bone Ca and P concentrations can reflect Ca and P homeostasis status, and their concentrations within normal range are essential for optimal bone mineralization and remodeling [15], thus their levels can represent bone health status. Studies have shown that laying hens with osteoporosis had the disorder of Ca and P homeostasis in serum [16,17]. In particular, 25-hydroxyvitamin D_3_ (25-OHD_3_) is a metabolite of vitamin D_3_ and it can transform to 1,25-dihydroxy-vitamin D_3_ (1,25-(OH)_2_D_3_), a key calcium regulation hormone, to promote bone mineralization. Serum 25-OHD_3_ concentration is associated with bone health, Ca and P metabolic utilization and homeostasis in poultry, and broilers with impaired bone development had low serum 25-OHD_3_ concentrations [17]. Based on the findings of above studies, therefore, we could speculate that the variation in keel bone characters reflected by changed serum bone turnover markers may affect the incidence of KBFs in laying hens by modulating keel bone metabolism and development.

However, based on our knowledge, there are limited studies that researched whether the keel bone character changes affect the occurrence of KBFs in laying hens in a longitudinal study. Thus, the primary objective of this study was to investigate the relationship between bone character changes before fractures and KBFs by determining several serum markers related to bone Ca and P metabolism and bone remodeling in laying hens housed in furnished cages. We hypothesized that abnormal bone metabolism and development could be a major factor inducing KBFs in laying hens.

## 2. Materials and Methods

### 2.1. Acquisition and Management of Animals

A total of 80 healthy Hy-Line Brown laying hens aged 18 weeks were purchased from a local poultry farm in Harbin, China. Laying hens were housed in 8 similarly furnished cages with 10 birds per cage. The birds in each cage were marked with different numerical leg-tags for easier identification. Each furnished cage was 150 cm × 70 cm × 70 cm and equipped with; (1) two wooden square perches of different heights, (2) an elevated closed-nesting box, (3) a rectangular feeder, and (4) a water-line with four nipple drinkers. The layout of equipment in the cages was similar to our previous description [18]. Moreover, the cages were placed in a semi-enclosed hen house with natural ventilation and a combination scheme of natural and artificial lights. Artificial light was programed for 16 h of light (5:30–21:30 h) and 8 h of darkness, and the light intensity was 18–22 lux. The temperature and relative humidity of laying hen house were 18–21°C and 55–70%, respectively. All birds were fed with a corn-soybean basal diet with 2787.8 kcal/kg metabolic energy and 16.40% crude protein, and they were free to feed and drink water during the entire experimental period from 18 WOA to 29 WOA. The ingredients and nutrient levels of basic diet fed to laying hens were shown in Table 1.

### 2.2. Experimental Design and Serum Sample Collection

**Figure 1:**
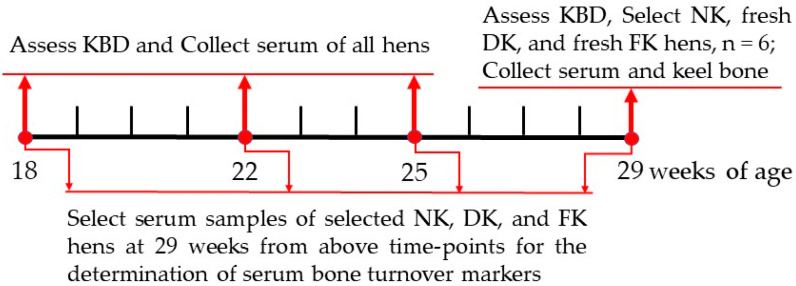
The experimental design, keel bone assessment, and sample collection time. NK = normal keel bone; DK = deviated keel bone; FK = fractured keel bone; KBD = keel bone damage.

Laying hens that developed keel bone damage (including fracture and deviation) at 29 WOA were used as focal animals for investigating the relationships between the incidences of keel bone damage and the changes of serum bone turnover markers in a longitudinal study. Blood samples of all laying hens were collected by wing vein puncture at four time-points: 18, 22, 25, and 29 WOA. Thereafter, the blood samples were centrifuged at 3500 rpm for 15 min, and the obtained serum samples were stored at -80°C for further analysis. Serum samples of the laying hens with normal keel (NK), deviated keel (DK) and fractured keel (FK) bones that occurred at 29 WOA were selected from all blood samples obtained at 18, 22, 25, and 29 WOA above time-points. Thus, the collection time-points of the blood sample for the focal animals were 11, 7, 4, and 0 weeks relative to 29 WOA. The experimental design, keel bone damage assessment, and sample collection time-points were displayed in Figure 1. Moreover, serum samples of the focal animals were used to determine the levels of bone turnover markers based on; (1) bone Ca and P metabolism and (2) osteoblast and osteoclast activity.

Keel bone status of all laying hens was assessed at 18, 22, 25, and 29 WOA using a portable X-ray instrument (WAT-LES100D, Shenzhen, China). Firstly, the hen was gently removed from its cage, one experimenter pulled the its legs caudally, while the other one fixed the wings above its back to ensure that the keel bone was not covered by its legs and wings. Then, the hen was placed the left side of its body on the surface of a digital detector panel of X-ray instrument, and the sagittal plane of the keel bone was at right angles to the detector plane of the X-ray instrument. Finally, the hen was slowly moved on the surface of detector panel to make sure that the images of X-ray evaluation of keel bone status were clear and accurate. The X-ray images of keel bone were analyzed based on the description of Eusemann et al. [8], and the NK, DK, and FK hens were marked according to the numbered leg-tags. The duration of X-ray evaluation, including the collection of hens, imaging, and returning the birds in their cages, took about three minutes per hen, and the evaluation process was performed by the two same experimenters at each time-point. Laying hens with NK, DK, and FK bones that occurred at 29 WOA were selected as focal animals for serum sample preparation. In the present study, a hen with DK and FK bone was considered to have FK. Therefore, there were 48 NK, 8 fresh DK, and 6 fresh FK hens at 29 WOA. Finally, all serum samples from 18 focal animals (*n* = 6 each group) per time-point were selected for bone character-related markers determination.

### 2.3. Keel Bone Sample Collection

At 29 WOA, 18 laying hens (*n* = 6 per group) were selected and slaughtered by cervical dislocation for keel bone sample collection. The keel bone was quickly excised from the body, and muscle and soft tissues that were attached to the bone were removed. Subsequently, the length from the caudal to the cranial tip and weight of each keel bone were measured using a digital caliper and an analytical balance, respectively, and 18 keel bone samples of the laying hens (*n* = 6 each group) were stored in the freezer at −80 °C until use.

### 2.4. Hematoxylin-Eosin (H&E) Staining

For each NK, DK, and FK bone, a 0.5 cm long bone piece was cut from approximately 2.5 cm from the caudal border of keel bone and used as bone sample, and the transverse plane of the piece was subjected to histological observation and analysis. The cut keel bone samples were fixed using 4% paraformaldehyde and decalcified with 10% ethylene diamine tetraacetic acid. After complete decalcification, each bone sample was embedded in paraffin and sliced at a thickness of 5 μm. Thereafter, the sliced sections were immersed into xylene solution followed by absolute alcohol with a concentration gradient in preparation for the H&E staining. The sections were dyed with hematoxylin for 8–10 min and differentiated with 1% hydrochloric acid alcohol. Afterwards, the sections were dehydrated using 85% and 95% alcohol for 5 min each and immersed into eosin for 8–10 min. After that, the sections were submerged in absolute alcohol followed by xylene with a concentration gradient for 5 min and sealed with neutral gum. Finally, the traeted sections were observed using optical microscopy (Nikon Eclipse E100, Nikon, Tokyo, Japan), and the pictures were taken under 100 times magnification.

### 2.5. Tartrate Resistant Acid Phosphatase (TRAP) Staining

The paraffin-embedded sections of each keel bone sample were dewaxed using xylene solution and ethyl alcohol with a concentration gradient for 5 min each. These sections were then incubated in distilled water at 37 °C for 2 h and hatched by the filtered TRAP staining solution (G1039, Servicebio, Wuhan, China) at 37 °C for 20 min. Subsequently, the sections were counterstained with hematoxylin for 15 s and differentiated with 1% hydrochloric acid alcohol. Thereafter, the sections were dehydrated using xylene for 5 min and were sealed with neutral balsam. Eventually, the sections were observed under an orthostatic light microscope (Nikon Eclipse E100, Nikon, Japan), and images were taken to analyze the staining results.

### 2.6. Determination of Serum Ca and P Metabolism-Related Markers

The concentration of serum Ca of the laying hens was measured using a microplate reader of the Ca assay kit (Kit number: C004-2-1), according to the manufacturer’s instruction (Nanjing Jiancheng Bioengineering Institute, Nanjing, China). Conversely, serum P concentration was determined by the molybdenum blue method using a serum P assay kit (Kit number: C006-1-1, Nanjing Jiancheng Bioengineering Institute, Nanjing, China). Consistently, the concentrations of serum 25-OHD_3_ (Kit number: JB278-Ch) and 1,25-(OH)_2_D_3_ (Kit number: JB331-Ch) (Shanghai Jinma Laboratory Equipment Corporation Ltd, Shanghai, China), parathyroid hormone (PTH) (Kit number: ML002803, Shanghai enzyme-linked Biotechnology Corporation Ltd, Shanghai, China), and calcitonin (CT) (Kit number: JB135-Ch, Shanghai Jinma Laboratory Equipment Corporation Ltd, Shanghai, China) were determined using the enzyme-linked immunosorbent assay (ELISA) method following the manufacturer’s instructions for the corresponding kits.

### 2.7. Determination of Serum Osteoblast and Osteoclast-Related Markers

The levels of bone formation (osteoblast activity) markers osteocalcin (OC) (Kit number: JB162-Ch) and ALP (Kit number: JB329X-Ch), and bone absorption (osteoclast activity) markers TRAP (Kit number: JB330X-Ch) and osteoprotegerin (OPG) (Kit number: JB163-Ch) in the serum of the laying hens were measured by ELISA method using a microplate reader of the corresponding ELISA kits (Shanghai Jinma Laboratory Equipment Corporation Ltd, Shanghai, China). The activity of serum corticosterone (CORT) was detected with an ELISA kit (Kit number: H205-1-2) following the manufacturer’s instruction (Nanjing Jiancheng Bioengineering Institute, Nanjing, China). A total of 50 microliters (μL) of serum sample (including 10 μL of serum and 40 μL of sample dilution) from each experimental sample were added into the micropore for physiological indicators evaluation. Moreover, a standard curve was obtained using the standard solutions of different concentrations for each marker to quantify the concentrations of the experimental samples. The optical density values were then measured at a wavelength of 450 nm using a microplate reader (Biotek Instrument Inc., Winooski, VT, USA).

### 2.8. Statistical Analysis

The collected data on bone characters were analyzed using the Statistic Package for Social Science software version 23.0 (SPSS 23.0; SPSS Inc., Chicago, IL, USA). The experimental unit of the present study was a single bird. Additionally, the data were tested for normality using the Kolmogorov-Smirnov test, and the data was found to be normally distributed. We performed a multivariate analysis of variance (ANOVA) using a general linear model with repeated measurements for body weight, serum markers related to Ca and P metabolism, and osteoblast and osteoclast activities based on the within-subjects factors Time (18, 22, 25, and 29 WOA) and between-subjects factors Group (NK, DK, and FK) and Cage (C1 to C8). This statistical analysis model included the effects of Time, Group, Cage, and their interaction, and therefore, it aided in determining whether bone character changes influence keel bone damage. In addition, the data on keel bone length and weight were analyzed using one-way ANOVA based on Duncan’s post hoc multiple range test. The results were presented as mean ± SEM, and the differences were considered statistically significant when *p* ≤ 0.05.

## 3. Results

### 3.1. Measurement of Bodyweight

The bodyweight of laying hens, with and without keel bone damage, at each time point, are shown in Table 2. The association between different sampling times (Time) and keel bone status (Group) and Cage had no significant effect on the bodyweight of the laying hens (*p* > 0.05). However, the bodyweight of laying hens was affected by Group (*p* < 0.05) and Time (*p* < 0.01). Additionally, the bodyweight of laying hens from all groups significantly increased with age (*p* < 0.05), while it was not different between 25 and 29 WOA (*p* > 0.05). The bodyweight of the laying hens in the FK group was significantly lower than in the NK group (*p* < 0.05). However, there was no significant difference in the bodyweight of the laying hens between the NK and DK groups (*p* > 0.05).

### 3.2. Measurement of Keel Bone Length and Weight

There were no significant differences in the length and weight of keel bones of the laying hens from three groups at 29 WOA (*p* > 0.05), as shown in Table 3.

### 3.3. Pathological Changes of Keel Bone

**Figure 2:**
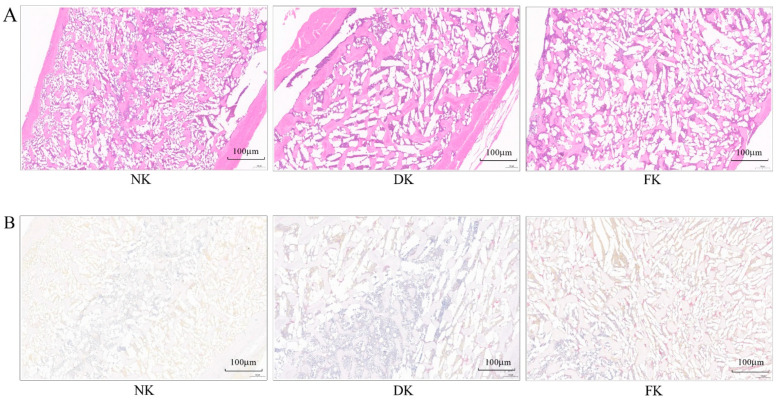
The results of hematoxylin-eosin (H&E) staining (**A**) and tartrate resistant acid phosphatase (TRAP) staining (**B**) of the keel bone in each group. NK = normal keel bone; DK = deviated keel bone; FK = fractured keel bone.

The results of H&E staining and TRAP staining of the keel bone in each group are shown in Figure 2. Figure 2A represents the result of H&E staining, which showed that the bone trabecular density was well-distributed, and the structural integrity was intact in the NK group. However, DK and FK groups exhibited evident bone structure changes with increased trabecular separation and reduced number relative to the NK group. In addition, Figure 2B displays the TRAP straining results of the keel bone, showing that the number of osteoclasts was relatively elevated in both DK and FK groups, with the FK group having the highest levels, compared with the NK group.

### 3.4. Determination of Serum Ca and P Metabolism-Related Markers

The concentrations of serum Ca and P metabolism-related markers of laying hens, from each group per time point, are shown in Table 4. The relationship between keel bone status (Group) and the testing time (Time) exhibited a significant effect on serum Ca, P, and 1,25-(OH)_2_D_3_ concentrations (*p* < 0.05) but had no significant impacts on serum 25-OHD_3_, PTH, and CT concentrations (*p* > 0.05) in the laying hens. Besides, the interaction between Group and Cage, Time and Cage, and Group and Time and Cage had no significant effect on serum Ca and P metabolism-related markers (*p* > 0.05).

As shown in Table 4, the concentration of Ca in the serum of laying hens was also influenced by Time (*p* < 0.01), and the serum Ca concentration of laying hens was significantly increased with the age from 18 WOA to 29 WOA (*p* < 0.05). However, serum Ca concentration of laying hens was not significantly influenced by Group (*p* > 0.05). The levels of P and PTH in the serum of laying hens were influenced by Time (*p* < 0.05) and Group (*p* < 0.05). The level of serum P was significant decreased with the age of laying hens (*p* < 0.05), while that of serum PTH was significant increased with the age of laying hens (*p* < 0.05). Meanwhile, the levels of P and PTH in serum of FK laying hens were significantly lower than that of NK and DK laying hens (*p* < 0.05), and their levels were not different between NK and FK laying hens (*p* > 0.05). The levels of 1,25-(OH)_2_D_3_, 25-OHD_3_, and CT in serum of laying hens were influenced by Group (*p* < 0.05), but not influenced by Time (*p* > 0.05). The levels of serum 1,25-(OH)_2_D_3_ and 25-OHD_3_ were significantly increased in FK laying hens compared to that in NK laying hens (*p* < 0.05). The DK laying hens had higher level of serum 1,25-(OH)_2_D_3_ and lower level of serum 25-OHD_3_ than those in NK laying hens (*p* < 0.05). Furthermore, the level of serum CT in DK laying hens was significantly higher than that in NK and FK laying hens (*p* < 0.05), but its level was not different between NK and FK laying hens (*p* > 0.05). Additionally, there was no significant difference in serum Ca and P metabolism-related markers between the cages (*p* > 0.05).

### 3.5. Determination of Serum Osteoblast and Osteoclast-Related Markers

The results of osteoblast and osteoclast-related markers in the serum of the laying hens are shown in Table 5 with a different keel bone status at each testing time-point. In this study, the interaction between keel bone status (Group), and testing time-points (Time), and Cage had no significant effect on serum osteoblast and osteoclast-related markers in laying hens (*p* > 0.05) except for the interaction between Group and Time which had a significant effect on the serum OC level (*p* = 0.05).

Moreover, the activities of ALP and TRAP, and the concentrations of OPG, OC, and CORT in serum of laying hens were also affected by Group (*p* < 0.01) and Time (*p* < 0.05). The activities of serum ALP and TRAP and the concentrations of serum OPG, OC, and CORT in the FK and DK laying hens were significantly increased compared to the NK laying hens (*p* < 0.05). The highest values of these bone turnover markers were found in FK laying hens, and the DK laying hens had generally intermediated values except for the level of serum OC, which was the highest in DK laying hens and was intermediated in FK laying hens (*p* < 0.05). There was a significant decrease in the activities of ALP and TRAP and the concentrations of OC and OPG in serum of laying hens with the increase of age from 18 WOA and 25 WOA (*p* < 0.05), and the lowest testing values of these markers were exhibited at 25 WOA compared to those at 18 and 22 WOA (*p* < 0.05). The levels of serum ALP and OC were significantly increased at 29 WOA compared to that at 25 WOA (*p* < 0.05), but the levels of serum TRAP and OPG were not overall different between 29 WOA and other time-points (*p* > 0.05). The serum CORT level was significantly higher at 29 WOA than that at 22 WOA (*p* < 0.05), but other than that, its level was not different between other testing time-points (*p* > 0.05). In addition, the cage factor had no significant effect on serum osteoblast and osteoclast-related markers in laying hens (*p* > 0.05).

## 4. Discussion

The keel is a structural bone necessary for essential activities and physiological processes in birds, such as flapping wings and providing ventilation for the lungs during inhalation and exhalation [19]. Thus, the health state of the keel bone influences the activity and health of birds. However, the recent ban of conventional cages in the European Union and other countries and the transition to alternative housing systems such as furnished cages, aviary, and cage-free systems make keel bone damage a severe health and welfare issue for the laying hens [2]. Numerous studies have reported that keel bone damage, especially KBFs, negatively affects the welfare and performance of laying hens by causing; (1) induced pain [20], (2) physiological stress [21], (3) fear and negative emotional state [22], (4) altered behaviors [23], and (5) reduced egg production [24] and quality [18]. In addition, severe KBFs could lead to the death of laying hens and cause additional economic losses for the laying hen production industries. Therefore, it is necessary to investigate the causes of keel bone damage, focusing on bone metabolism and development. Consequently, we designed and performed this experiment to evaluate the serum bone parameters changes in laying hens.

The bodyweight of laying hens from all groups increased with age, however, the FK hens had an observably lowed bodyweight compared to NK hens. This finding indicates that the occurrence of KBFs may be associated with the changes in the body weight of laying hens. Moreover, previous studies found that the bodyweight of laying hens affected the keel bone quality and vice versa [25,26], and the laying hens with fractured keel bone generally had a lighter bodyweight than those with a normal keel bone [6,18]. This finding was similar to our observation that the bodyweight of FK laying hens was lower than NK hens and reflects that the bodyweight is related to KBFs in laying hens. The present study results also showed that the length and weight of keel bones were not significantly different in NK, DK, and FK laying hens; therefore, suggesting that keel bone damage might not impair the bone growth of the hens housed in the furnished cages.

In poultry, bone metabolism and health involve multiple regulated factors. Ca and P are two essential elements associated with the mineralization and mechanical strength of bones in laying hens [27]. Many researchers demonstrated that the levels of serum Ca and P reflects the bone health status [15,28], and a reduction in the concentrations of Ca and P in serum was observed when chickens had bone disease with abnormal metabolism caused by dietary Ca and P deficiency [16,29]. This discovery implies the possibility of bone health impairment through the modulation of serum Ca and P levels. In the present study, serum Ca concentration in laying hens from 18 WOA to 29 WOA was increased, and its level was not statistically different between groups, but FK hens had a significant lower serum Ca level at 25 and 29 WOA than NK hens. While the level of serum P decreased with age, and FK laying hens had a significantly low serum P level compared NK hens, it suggested that there was a significant relationship between the homeostasis of serum Ca and P and keel bone fractures, but not keel bone deviations. Moreover, before the keel bone fractures, the concentrations of serum Ca in FK laying hens increased at 22 WOA and decreased at 25 WOA compared to those in NK laying hens. This finding indicates that abnormal serum Ca changes could be involved in bone fractures. An active metabolite of vitamin D_3_, 25-OHD_3_, is hydroxylated via the liver and plays important regulatory roles in the metabolic utilization and homeostasis of Ca and P and maintaining bone health in animals [30]. Another active form of vitamin D_3_ is 1,25-(OH)_2_D_3_, produced by 25-OHD_3_ through the activation of 25-(OH)_2_-1-a-hydroxylase in the kidney. It mainly promotes Ca absorption in the gut. Accordingly, some studies reported that bone damage is related to the variation in serum 25-OHD_3_ and 1,25-(OH)_2_D_3_ and low levels of serum 25-OHD_3_ generally resulted in poor bone health in broilers [17]. Low levels of serum 25-OHD_3_ were also reported to increase the risk of bone fractures in humans [31]. In this study, no significant differences in serum 25-OHD_3_ and 1,25-(OH)_2_D_3_ levels were reported in three groups across the testing time-points as an entity. However, there were differences in serum 25-OHD_3_ and 1,25-(OH)_2_D_3_ levels between the groups. The FK laying hens had significantly higher serum 25-OHD_3_ and 1,25-(OH)_2_D_3_ levels than NK and DK laying hens, indicating that bone health status was associated with these indexes’ levels but not the age of the birds. Thus, our results are inconsistent with the previous findings [17,31], which may be due to the species type used.

Commercial laying hens designated for high egg production have a special demand for Ca necessary for eggshell formation, and approximately 3 g of Ca is required to synthesize an eggshell [32]. The eggshell formation is a rapid process of mineralization in laying hens. During the active phase of mineralization, Ca deficiency-induced hypocalcemia stimulates PTH synthesis, increasing the level of 1,25-(OH)_2_ D_3_ [33], thus enhancing the circulating ionic Ca levels and promoting bone resorption. It was approved that bone metabolism is affected by several factors, such as PTH and CT [34,35]. In this study, the levels of serum PTH in laying hens at 25 WOA was significantly higher than that at 22 WOA, and the serum PTH level in laying hens from the DK group was significantly elevated compared to NK group, but no significant difference was found between the NK and FK groups. These results might indicate that increased serum PTH stimulates the productions of 1,25-(OH)_2_D_3_ and Ca in blood. Thus, PTH levels could be related to abnormal bone metabolism with accelerated bone resorption, resulting in bone damage. Additionally, when the amount of diet-derived Ca received by laying hens is insufficient for eggshell formation and high egg production, Ca deficiency-induced hypocalcemia could further stimulate PTH production. This phenomenon enhances calcium-regulating hormone 1,25-(OH)_2_D_3_ levels, thus increasing Ca amounts for eggshell formation [34]. Meanwhile, it was reported that CT inhibits osteoclast activity and reduces bone resorption by rapidly decreasing Ca levels in the blood [36]. In this study, NK and FK laying hens had elevated serum Ca levels, while DK birds had increased serum CT. However, the CT levels in FK birds were not significantly different from that of NK birds. This observation demonstrated that increased serum CT levels in laying hens with impaired keel bone might reduce serum Ca levels, thereby reducing bone resorption and regulate routine bone metabolism.

Bone homeostasis is maintained by a balance between bone resorption by osteoclasts and bone formation by osteoblasts. The imbalance in bone remodeling leads to bone diseases, such as osteoporosis and osteopetrosis [37]. ALP and OC are involved in bone formation and mineralization, and their levels reflect osteoblast activity and bone metabolism status [38]. Consequently, previous studies showed that serum ALP and OC levels were consistently increased when bone formation and osteoblast activity were elevated [14,39]. Moreover, TRAP is an indicator of osteoclast activity and bone resorption. Several studies reported that the TRAP level was elevated when the bone mass loss was associated with the reduced trabecular number and bone damage [14,40], suggesting increased osteoclast activity and bone resorption. In addition, bone TRAP staining, one of the bone resorption markers, can identify osteoclasts [41]. In the present study, the ALP, OC, and TRAP levels in serum of laying hens were decreased with age from 18 WOA and 25 WOA, and their levels were increased at 29 WOA. However, laying hens with keel bone damage had overall elevated serum ALP, OC, and TRAP levels throughout the trial and relatively increased osteoclasts numbers at 29 WOA compared to the laying hens with normal keel bone. This finding suggested a possible association between keel bone damage and abnormal bone metabolism. In addition, OPG has been identified as a major cytokine regulating bone metabolism [42]. OPG is a decoy receptor of the tumor necrosis factor receptor family that binds to the receptor of the activator of the nuclear factor κB-ligand (RANKL), thus inhibiting the activity of RANKL. Once OPG binds to RANKL, osteoclast formation, activation, and survival cease rapidly, stimulating the initiation of subsequent bone formation [42,43]. Consistently, it was reported that serum OPG level was higher in osteoporotic patients than in healthy individuals, indicating that increased OPG production could suppress bone resorption [44]. In this study, the levels of serum OPG in DK and FK laying hens, especially in the FK birds, were higher than in NK hens, implying that increased serum OPG levels in laying hens with keel bone damage might prevent bone resorption and maintain normal bone metabolism.

In this study, overall, abnormal bone metabolism could cause keel bone damage in laying hens, which was mainly indicated from the results detected at 18, 22, and 25 WOA but not at 29 WOA because the effect at 29 WOA could be a result of the keel bone damage, rather than causal. Additionally, although the current study suggested that keel bone damage could be associated with abnormal bone metabolism reflected by changed serum markers in relation to bone turnover in laying hens, this study had limitations, such as the younger hens, a shorter experimental period, a smaller sample size, and the lack of the analysis of bone mineralization markers and serum RANKL level, as well as the age at onset of lay and the egg production of laying hens in each group. Previous studies reported that these factors could affect the development of KBFs in laying hens [26,45]. Besides, one of the most important limitations is the young age of the laying hens. In the present study, the laying hens at 18 WOA were used and studied for 11 weeks, but the keel bone was incompletely ossified and the incidence of KBFs was relatively low during this period. Thus, further study regarding this should be considered for these causative factors.

## 5. Conclusions

In conclusion, this study shows that DK and FK laying hens, especially the FK hens, had significantly lighter bodyweight than the NK hens. The study also reports that the levels of serum markers related to Ca and P metabolism and osteoblast and osteoclast activity varied with age in DK and FK hens (with more variation in FK hens) compared to the NK hens. Additionally, as reflected by bone H&E staining, DK and FK laying hens had changed bone structure compared to NK hens. These results imply that abnormal bone metabolism, depicted by the varying levels of serum bone turnover markers and bone staining, could be related to keel bone damage (especially fractures) in laying hens. Thus, this study postulates that abnormal bone metabolism is a causative factor of keel bone damage in caged laying hens.

## Figures and Tables

**Table 1:** Ingredients and nutrient levels of laying hen diets.

Ingredients (%)		Nutrients	
Corn (%)	62.5	Metabolic energy (Kcal/kg)	2787.8
Soybean meal	24.0	Crude protein (g/kg)	16.40
Wheat bran	2.50	Lysine (g/kg, measured)	8.81
Limestone	8.15	Methionine (g/kg, measured)	3.39
Calcium hydrogen phosphate	1.37	Calcium (g/kg)	33.4
Sodium chloride	0.33	Calcium (g/kg, measured)	34.6
DL-Methionine	0.15	Total phosphorus (g/kg)	5.88
Vitamin-mineral premix ^1^	1.00	Total phosphorus (g/kg, measured)	4.98
Total	100	Available phosphorus (g/kg)	3.66
		Available phosphorus (g/kg, measured)	3.70
		Sodium (g/kg, measured)	1.16
		Magnesium (g/kg, measured)	2.22
		Manganese (mg/kg, measured)	139.1

^1^ Vitamin-mineral premix that provided the following per kilogram of mixed feed: Manganese, 50.18 mg; Zine, 40.22 mg; Iron, 40.14 mg; Copper, 6.10 mg; Iodine, 0.29 mg; Selenium, 0.18 mg; vitamin A, 1,2500 IU; vitamin D3, 3750 IU; vitamin E, 15 IU; vitamin K3, 2.5 mg; vitamin B1, 2.5 mg; vitamin B2, 7.0 mg; vitamin B6, 3.75 mg; vitamin B12, 0.015 mg; folic acid, 1.0 mg; pantothenic acid, 12.5 mg; niacin, 25 mg; and biotin, 0.075 mg.

**Table 2:** Bodyweight changes of laying hens with age in three groups.

Main Effect	BW (kg)	Main Effect	BW (kg)	Main Effect	*p*-Value
Time (week)		Cage (No)		Time	0.001
18	1.51 ^c^	C1	1.99	Group	0.046
22	1.96 ^b^	C2	1.86	Cage	0.394
25	2.01 ^a^	C3	1.81	Time × Group	0.874
29	2.07 ^a^	C4	1.87	Group × Cage	0.235
SEM	0.03	C5	1.92	Time × Cage	0.989
Group		C6	1.85	Group × Time × Cage	0.906
NK	1.92 ^x^	C7	1.83		
DK	1.87 ^x,y^	C8	1.75		
FK	1.79 ^y^	SEM	0.05		
SEM	0.03				

NK = normal keel bone; DK = deviated keel bone; FK = fractured keel bone. ^a,b,c^ Means with different superscripts within the same column represent significant difference between times (*p* < 0.05); ^x,y^ Means with different superscripts within the same column represent significant difference between groups (*p* < 0.05); means with the same or no superscripts represent no significant difference (*p* > 0.05). All results were expressed as mean and a pool SEM, *n* = 6 for each group per time-point.

**Table 3:** Keel bone length and weight of laying hens in three group.

Item (Unit)	Group			*p*-Value
	NK	DK	FK	
Keel bone length (mm)	104.49 ± 1.17	104.69 ± 1.40	101.81 ± 1.84	0.339
Keel bone weight (g)	8.39 ± 0.24	7.61 ± 0.32	7.67 ± 0.49	0.276

NK = normal keel bone; DK = deviated keel bone; FK = fractured keel bone. All results were expressed as mean and SEM, *n* = 6 each group.

**Table 4:** Measurement of serum Ca and P metabolism-related markers in laying hens.

Main Effect	Treatment	Ca	P	1,25-(OH)_2_D_3_	25-OHD_3_	PTH	CT
		(mmol/L)	(mmol/L)	(pg/mL)	(ng/mL)	(ng/L)	(ng/L)
Time (week)							
	18	1.47 ^c^	2.45 ^a^	132.53	8.16	40.38 ^ab^	84.77
	22	1.76 ^b^	2.28 ^ab^	132.80	8.28	39.47 ^b^	84.55
	25	1.89 ^ab^	2.12 ^b^	130.33	8.21	40.80 ^a^	84.87
	29	1.93 ^a^	2.02 ^b^	132.32	8.31	40.23 ^ab^	83.46
	SEM	0.06	0.10	1.03	0.06	0.34	0.59
Group							
	NK	1.80	2.36 ^x^	119.40 ^z^	8.11 ^y^	39.37 ^y^	78.90 ^y^
	DK	1.77	2.31 ^x^	134.05 ^y^	7.65 ^z^	42.17 ^x^	95.27 ^x^
	FK	1.72	1.98 ^y^	142.38 ^x^	8.96 ^x^	39.12 ^y^	79.07 ^y^
	SEM	0.05	0.09	0.89	0.05	0.29	0.51
Cage (No)							
	C1	1.67	2.58	128.41	7.89	40.35	88.03
	C2	1.68	2.34	138.24	8.40	40.82	87.23
	C3	1.68	2.41	130.92	8.62	39.34	78.63
	C4	1.77	2.11	127.02	7.97	40.67	87.17
	C5	1.83	2.06	132.00	8.14	39.89	84.12
	C6	1.86	2.28	128.06	7.98	41.31	86.13
	C7	1.81	2.16	131.79	8.26	40.35	84.35
	C8	1.71	1.90	139.62	8.92	38.99	79.83
	SEM	0.06	0.08	0.89	0.07	0.56	0.64
*p*-value							
	Time	0.004	0.016	0.100	0.229	0.046	0.202
	Group	0.466	0.016	<0.001	<0.001	0.007	<0.001
	Cage	0.318	0.051	0.514	0.328	0.924	0.535
	Time × Group	0.047	0.024	0.045	0.158	0.557	0.284
	Group × Cage	0.064	0.712	0.251	0.467	0.938	0.478
	Time × Cage	0.501	0.197	0.160	0.424	0.704	0.196
	Group × Time × Cage	0.399	0.097	0.126	0.266	0.514	0.255

Ca = calcium; P = phosphorus; 1,25-(OH)_2_D_3_ = 1,25-dihydroxyvitamin D_3_; 25-OHD_3_ = 25-hydroxyvitamin D_3_; PTH = parathyroid hormone; CT = calcitonin; NK = normal keel bone; DK= deviated keel bone; FK = fractured keel bone. ^a,b,c^ Means with different superscripts within the same column represent significant difference between time-points (*p* < 0.05); ^x,y,z^ Means with different superscripts within the same column represent significant difference between groups (*p* < 0.05); means with the same or no superscripts represent no significant difference (*p* > 0.05). All results were expressed as mean and a pool SEM, *n* = 6 for each group per time-point.

**Table 5:** Measurement of serum bone metabolism and remodeling-related markers in laying hens.

Main Effect	Treatment	ALP	OC	TRAP	OPG	CORT
		(U/L)	(ng/L)	(U/L)	(ng/L)	(ng/L)
Time (week)						
	18	2049.87 ^a^	8.73 ^ab^	170.53 ^ab^	497.70 ^ab^	429.26 ^ab^
	22	1987.69 ^b^	8.86 ^a^	172.38 ^a^	507.01 ^a^	424.75 ^b^
	25	1963.06 ^b^	8.55 ^b^	167.03 ^b^	491.57 ^b^	433.37 ^ab^
	29	2041.16 ^a^	8.94 ^a^	169.29 ^ab^	494.28 ^b^	436.81 ^a^
	SEM	19.89	0.07	1.93	7.70	4.39
Group						
	NK	1887.74 ^z^	7.72 ^z^	154.55 ^z^	475.17 ^z^	397.79 ^y^
	DK	2036.21 ^y^	9.47 ^x^	167.90 ^y^	489.61 ^y^	399.69 ^y^
	FK	2107.40 ^x^	9.12 ^y^	186.97 ^x^	528.14 ^x^	500.66 ^x^
	SEM	17.22	0.06	1.67	6.67	3.80
Cage (No)						
	C1	1960.28	8.67	159.48	489.55	398.36
	C2	2027.19	9.23	177.64	517.66	449.24
	C3	1958.93	8.35	168.68	507.71	449.54
	C4	1939.33	8.56	160.74	485.53	401.55
	C5	2023.58	8.83	169.90	500.03	434.09
	C6	1968.39	8.56	160.97	469.64	399.78
	C7	2036.20	8.76	172.10	488.85	431.71
	C8	2150.20	9.19	187.75	525.34	497.23
	SEM	29.83	0.11	2.89	11.55	6.59
*p*-value						
	Time	0.023	0.014	0.048	0.042	0.045
	Group	0.001	< 0.001	< 0.001	0.009	<0.001
	Cage	0.207	0.654	0.743	0.529	0.679
	Time × Group	0.222	0.050	0.200	0.894	0.589
	Group × Cage	0.346	0.197	0.745	0.992	0.247
	Time × Cage	0.318	0.206	0.267	0.925	0.664
	Group × Time × Cage	0.306	0.160	0.467	0.911	0.636

ALP = alkaline phosphatase; TRAP = tartrate-resistant acid phosphatase; OPG = osteoprotegerin; OC = osteocalcin; CORT = corticosterone; NK = normal keel bone; DK= deviated keel bone; FK = fractured keel bone. ^a,b,c^ Means with different superscripts within the same column represent a significant difference between time-points (*p* < 0.05); ^x,y,z^ Means with different superscripts within the same column represent a significant difference between groups (*p* < 0.05); means with the same or no superscripts represent no significant difference (*p* > 0.05). All results were expressed as mean and a pool SEM, *n* = 6 for each group per time-point.

## Data Availability

The data presented in this study are available on reasonable request from the corresponding authors.
